# A naked-eye biosensing system based on one-pot RPA-CRISPR/Cas12a driver G4-hemin self-assembly for *Mycobacterium tuberculosis*

**DOI:** 10.3389/fchem.2025.1631086

**Published:** 2025-08-07

**Authors:** Ting Yuan, Jianbo Yuan, Jian Huang, Nana Li, Huan Cai, Yan Yang, Juan Li, Rui Chen, Xun Min

**Affiliations:** ^1^ Department of Laboratory Medicine, Affiliated Hospital of Zunyi Medical University, Zunyi, China; ^2^ School of Laboratory Medicine, Zunyi Medical University, Zunyi, China; ^3^ Key Laboratory of Clinical Laboratory Diagnostics (Ministry of Education), College of Laboratory Medicine, Chongqing Medical University, Chongqing, China; ^4^ Department of endocrinology, Affiliated Hospital of Zunyi Medical University, Zunyi, China

**Keywords:** RPA-CRISPR/Cas12a, G4-hemin DNAzyme, naked-eye biosensing system, one-pot, *Mycobacterium tuberculosis*

## Abstract

**Introduction:**

The rapid and accurate identification of *Mycobacterium tuberculosis* (MTB) is essential for effective tuberculosis (TB) control. However, conventional diagnostic methods for MTB suffer from limitations such as low sensitivity, poor specificity, high cost, reliance on specialized instruments, and complex, time-consuming procedures. To address these challenges, there is an urgent need for a simple, rapid, and highly sensitive detection method that can be deployed in point-of-care settings.

**Methods:**

We developed a one-pot biosensing system combining recombinase polymerase amplification (RPA) and CRISPR/Cas12a-driven G4-hemin self-assembly for the colorimetric detection of MTB. Glycerol was employed as a phase-separation barrier to prevent interference between RPA amplification and CRISPR/Cas12a trans-cleavage. A single-stranded DNA (ssDNA) probe, designed to self-assemble with ssDNA-hemin into G4-hemin nanozymes upon CRISPR/Cas12a-mediated cleavage, served as the reaction substrate. The ssDNA-hemin further enhanced the catalytic activity of the generated G4-hemin DNAzyme. The entire assay was completed in a single step within 60 min without requiring complex instrumentation.

**Results and Discussion:**

Under optimized conditions, the biosensing system achieved ultrasensitive naked-eye detection of MTB with a limit of detection (LOD) of 10 copies/μL, comparable to traditional four-step fluorescent assays. Clinical validation using 104 patient samples demonstrated high concordance with standard diagnostic methods. This approach combines the advantages of recombinase polymerase amplification (RPA), CRISPR/Cas12a specificity, and G4-hemin DNAzyme-based colorimetric signal amplification, enabling simple, equipment-free visual detection. Given its speed, sensitivity, and ease of use, this biosensing system holds significant promise for point-of-care MTB nucleic acid testing in resource-limited settings.

## 1 Introduction

*Mycobacterium tuberculosis* (MTB) is the causative agent of tuberculosis (TB) and the leading cause of death from a single infectious pathogen worldwide (Afriyie-Asante et al., 2021; Tu et al., 2019). Its widespread prevalence, lengthy treatment, and high fatality rate make TB a critical global health challenge (Marx et al., 2020). The conventional culture method for acid-fast bacilli is the diagnostic gold standard, but it is time-consuming and expensive, often resulting in delays in diagnosis (Isa et al., 2018; Lau et al., 2015). As a result, clinicians may begin anti-TB therapy based on clinical judgment alone, which can contribute the emergence of drug-resistant strains (Massud et al., 2022). Sputum smear microscopy has low sensitivity, missing many cases due to insufficient bacterial load in samples (Babin et al., 2021; Yang et al., 2020). The interferon-γ release assay (IGRA) is an *in vitro* immunological test recommended by the WHO for MTB infection (Mendelsohn et al., 2021; Zaia, 2022). However, IGRA results must be interpreted in conjunction with clinical symptoms, imaging, and other examinations, and it cannot independently confirm TB infection (Harling et al., 2019). Xpert MTB/RIF, a fully automated real-time PCR assay for MTB and rifampicin resistance, offers improved sensitivity but requires specialized equipment and trained personnel, limiting its utility in resource-limited settings (Dhar et al., 2020; Kohli et al., 2021; Turner et al., 2020). Consequently, there is an urgent need for an innovative point-of-care testing (POCT) method for MTB that provides high sensitivity and specificity, is rapid and affordable, and does not require complex instrumentation.

Clustered Regularly Interspaced Short Palindromic Repeats (CRISPR) and CRISPR-associated proteins (Cas) are components of the prokaryotic adaptive immune system (Aulicino et al., 2022; Krysler et al., 2022; Li W. et al., 2022). In this system, a Cas nuclease is guided by a single-guide RNA (sgRNA) to a complementary DNA sequence, thereby preventing the integration of foreign genetic elements into the bacterial genome (Rabinowitz et al., 2020; Roy et al., 2020). Cas12a, a type V CRISPR effector, uses CRISPR RNAs (crRNAs) to recognize targets in single-stranded or double-stranded DNA (ssDNA or dsDNA) (Guo et al., 2019; Shebanova et al., 2022). Once Cas12a, crRNA, and target DNA form a ternary complex, Cas12a exhibits nonspecific trans-cleavage activity, cutting any single-stranded DNA reporters in the reaction and thereby generating a detectable signal (e.g., fluorescence) (Curti et al., 2020; Liang et al., 2019; Lu et al., 2020). Coupling this trans-cleavage activity with an isothermal amplification like recombinase polymerase amplification (RPA) greatly enhances detection sensitivity (Hang et al., 2022; Kozlowski et al., 2021). However, RPA-CRISPR-based methods usually require a two-step reaction, making the operation cumbersome and prone to cross-contamination (Li F. et al., 2022). Meanwhile, the signal output mode of RPA-CRISPR is usually fluorescence (Fozouni et al., 2021; Huang et al., 2021). Interpretation of results often requires specialized equipment, and the workflow involves multiple steps, which is cumbersome and not ideal for POCT of infectious diseases. Therefore, the one-step visualisation method based on RPA-CRISPR, which does not require special instrumentation, still has a huge scope for exploration.

G-quadruplex (G4) structures are four-stranded DNA or RNA configurations formed by sequences rich in tandem guanine repeats (Chen et al., 2021; Li J. et al., 2021; Piazza et al., 2010). G4 structures play essential roles in various biological processes, including telomere maintenance, DNA replication, transcription, and translation of certain genes (De Magis et al., 2020). G4-based aptamers have been widely used for molecular recognition, such as detecting proteins and capturing specific targets (e.g., insulin, IGF-II), as well as in drug delivery for photodynamic therapy. G4 DNAzymes (DNA enzymes formed by G4-hemin complexes) have been developed for diverse biosensing applications (Kumar et al., 2020). For instance, Tsang et al. reported a low-molecular-weight G4 DNAzyme by combining a G-quadruplex with hemin to replace traditional enzymes in a chemiluminescent assay, and Wang et al. described a G4/hemin-based colorimetric method to detect *Bacillus anthracis* (Wang et al., 2022). Despite these advances, significant challenges remain in improving the efficiency of G4-hemin DNAzyme catalysts (Chen et al., 2017; Liu et al., 2014; Wang et al., 2016).

To address the above challenges, we developed a GOR-CRISPR colorimetric assay (Glycerol-enhanced One-pot RPA-CRISPR/Cas12a with G4-hemin DNAzyme). Firstly, to solve the problem of the RPA-CRISPR/Cas12a one-step method, we introduced glycerol, which acts like a barrier to separate the RPA and CRISPR/Cas12a systems. When enough RPA products are produced, the products are able to initiate the CRISPR/Cas12a reaction through glycerol. Thus, the sensitivity and operation simplicity of the integrated system are improved. Secondly, Visual, easy-to-interpret signal outputs made possible by the introduction of G4-hemin self-assembly nanozyme. Thirdly, the catalytic activity of G4-hemin self-assembly nanozyme is significantly higher than that of conventional G4-hemin DNAzyme, resulting in a substantial increase in sensitivity. More importantly, current methods based on reporter G4-hemin DNAzyme require multi-step reactions and operations, limiting their wide application (Obi et al., 2020). Thus, device-free, visual, easy-to-use and highly sensitive detection of *Mycobacterium tuberculosis* was achieved by cleverly integrating GOR-CRISPR and G4-hemin DNAzyme. The entire identification process is completed within 60 min, with a detection limit of 10 copies/μL and excellent specificity and sensitivity. We validated the platform by detecting MTB in clinical samples, demonstrating that this one-step RPA-CRISPR/Cas12a system with glycerol enables highly sensitive, visual detection of MTB DNA. In summary, our study establishes a foundation for a rapid and portable MTB diagnostic method suitable for future POCT applications.

## 2 Materials and methods

### 2.1 Materials and reagents

The sequences of all synthesized DNA or RNA, synthetic targets, RPA primers, Cas12a-crRNA, and fluorescent reporters used in this study are listed in Supplementary Table S1. The MTB IS1081 insertion element (Gene ID: 32286187, sequence dated 2020-04-24) was synthesized by Sangon Biotech (Shanghai, China). The MTB IS6110 insertion element (GenBank: X17348, updated 2020-04-24), along with genomic DNA from *Haemophilus influenzae*, *Streptococcus pneumoniae*, *Klebsiella pneumoniae*, *Pseudomonas aeruginosa*, and *Mycoplasma pneumoniae*, were used for specificity tests. LbaCas12a (Cpf1, M0653T) and NEBuffer 2.1 (B6002S) were purchased from New England Biolabs (United States). The lyophilized TwistAmp Basic kit (product code: TABAS03KIT, TwistDx, United Kingdom), containing the core reaction mix of recombinase, single-stranded DNA-binding protein, and strand-displacing polymerase, was used for RPA reactions. 3,3′,5,5′-Tetramethylbenzidine (TMB) and 30% H_2_O_2_ were obtained from Shanghai Aladdin Biotech (China). The TMB substrate solution was purchased from Beyotime Biotechnology (Shanghai, China). Ultrapure deionized water (18.2 MΩ cm), obtained from a Millipore Milli-Q system, was used throughout the study. MTB genomic DNA samples (for clinical evaluation) were provided by the Affiliated Hospital of Zunyi Medical University (China). Atomic force microscopy (AFM) images of G4-DNA-hemin, ssDNA-hemin, dsDNA-hemin, and ssDNAR were obtained using a Bruker Dimension Icon AFM (nominal spring constant ∼5.0 N/m, force-separation indentation speed 150 nm/s).

### 2.2 Glycerol-enhanced one-pot RPA-CRISPR Cas12a analysis

RPA has replaced traditional PCR methods to reduce detection time and decrease reliance on electrically powered devices. The RPA primer design followed the guidelines provided in the Manual of Assay Design included in the TwistAMP Basic KIT. To enhance the sensitivity and speed of the amplification process, the target size of the amplified product was set between 90 and 150 bp. Primers with lengths of 32–38 nucleotides and GC levels varying from 20% to 60% were meticulously constructed to avoid any notable palindromes or complementary sequences. Detailed information on the reaction system and RPA primers is presented in Supplementary Table S1. The one-pot setup was prepared in two parts, designated A and B. Part A consisted of NEBuffer (1 × 2.1r), crRNA (1 μM), ssG4R (2 μM), LbCas12a (1 μM), and 15% glycerol, while part B comprised the RPA system with primer-free rehydration buffer (29.5 μL), a single freeze-dried reaction pellet, forward and reverse primers (240 nM each), the target sample, and RNase-free water. The final 20 μL one-pot solution was achieved by adjusting the concentrations of each element. In the one-pot procedure, the process began with the careful introduction of 10 μL of solution A at the bottom of the tube. Following this initial step, 9.5 μL of solution B was added into the well of the tube. Subsequently, a drop of magnesium acetate, measured at 0.5 μL and at a concentration of 280 mM, was introduced into the mixture. To ensure thorough mixing, the tube was then briefly centrifuged. Once the components were adequately combined, the fluorescence signal was closely monitored for any changes. During the one-pot RT-RPA-CRISPR/Cas12a tests, it was noted that most of the components used were similar to those included in the original one-pot setup. This consistency in the materials used underscores the reliability of the procedural setup. All reactions were conducted at a controlled temperature of 37 °C and were allowed to proceed for a duration of 60 min, ensuring optimal conditions for the reactions to take place.

### 2.3 Design the DNA nanozyme color reaction

This research presents a newly created visualization system that makes use of the peroxidase activity associated with a G-quadruplex-hemin DNA nanozyme. The color reaction was based on the DNA nanozyme, ssDNA-hemin (sequence: hemin-TGGGTAGGGCGGGTTGGGAAA-3′). Utilizing the Cas12a reaction method described previously, the reverse complementary sequence ssDNAR (Wang et al., 2022) (5′-TTT​CCC​AAC​CCG​CCC​TAC​CCA-3′) was included as a reporter probe in the Cas12a detection system. A mixture of the Cas12a reaction product (20 μL) and 20 μM ssDNA-hemin (2 µL) was heat treated for 5 min at 95 °C, followed by cooling to room temperature to facilitate annealing of the DNA nanozyme (G4-DNA-hemin) and probe (ssDNAR). Subsequently, 100 μL of 3,3,5,5-tetramethylbenzidine (TMB) was introduced; the emergence of a clear blue hue after 5 min signified the presence of the target DNA, while solutions lacking the target DNA were either colorless or exhibited a pale blue tint.

### 2.4 Statistics

Statistical significance was set at not significant (NS), *P* > 0.05; *, *P* ≤ 0.05; **, *P* ≤ 0.01; ***, *P* ≤ 0.001; and ****, *P* ≤ 0.0001. All statistical analyses were performed using the Origin Prism software.

### 2.5 Design and amplification of RPA primers and crRNAs

The MTB-DNA sequence was inserted into a specially engineered pUC57 vector, and the resulting plasmid used as a template for RPA amplification and the CRISPR/Cas12a assay. Oligonucleotide primers for RPA were obtained from Sangon Biotech. Primer candidates were initially screened using the TwistAmp Basic kit according to the manufacturer’s instructions in 50 μL reactions. After identifying the optimal primer sequences, lyophilized primer pellets were prepared for use in TwistAmp Exo reactions. Each RPA reaction (50 μL final volume) contained 2.4 μL of forward primer (10 μM), 2.4 μL of reverse primer (10 μM), 29.5 μL of rehydration buffer (from the TwistAmp kit), 2.5 μL of MgOAc (280 mM), 2 μL of extracted MTB genomic DNA (∼500 ng), and nuclease-free water to volume. Based on the MTB genome, the 17 bases immediately following the protospacer adjacent motif (PAM, TTTN/TTN) in the IS1081 and IS6110 sequences were chosen as candidate target sites for species identification. Two MTB genetic targets (IS1081 and IS6110) were used to evaluate crRNA efficiency and specificity (see Supplementary Table S1 for sequences). Each crRNA comprised a 22-nucleotide target-specific sequence plus the constant repeat region required for Cas12a binding.

## 3 Results and discussion

### 3.1 Principle of the GOR-CRISPR with G4-hemin DNAzyme biosensor

**FIGURE 1:**
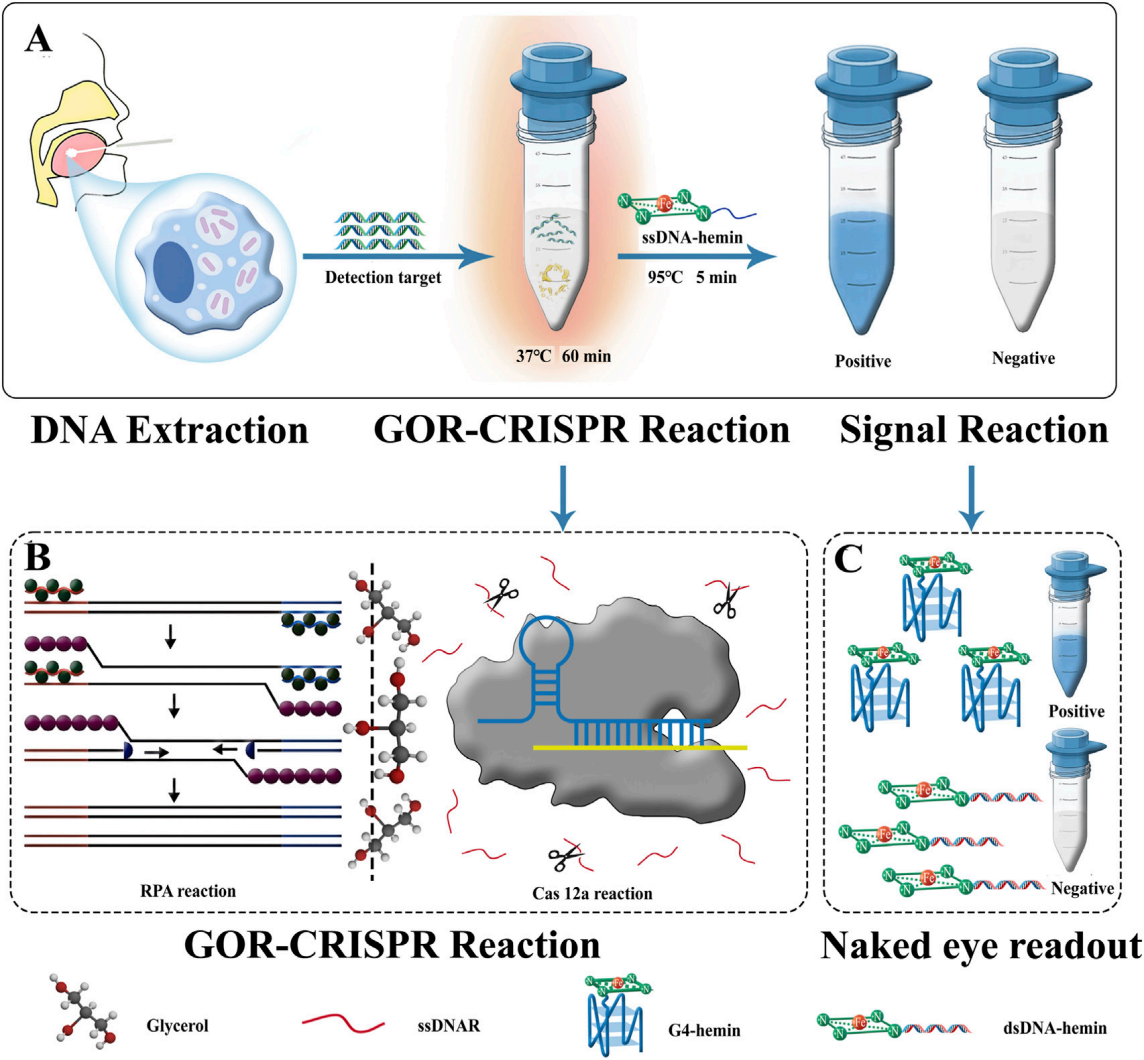
Schematic of GOR CRISPR reaction and G4-hemin DNAzyme visualization system for MTB detection. **(A)** Flow of application the of GOR CRISPR reaction and G4-hemin DNAzyme visualization system. **(B)** Schematic of the GOR CRISPR reaction. **(C)** Naked eye readout: the Cas12a reaction is supplemented with ssDNAR antisense DNA as a detection probe. SsDNA-hemin is introduced following cleavage of ssDNAR by Cas12a. A complex consisting of cleaved ssDNAR fragments and ssDNA-hemin can be formed, catalyzing TMB oxidation and turning the solution blue. Without Cas12a cleavage of the ssDNAR, ssDNA-hemin forms a complementary pair with its antisense molecule ssDNAR, resulting in the formation of dsDNA. The absence of enzyme activity in dsDNA-hemin causes the solution to remain colorless. Within 60 min, the whole detection procedure can be completed.

We integrated the catalytic activity of G4-hemin DNAzyme with a glycerol-enhanced one-pot RPA-CRISPR/Cas12a reaction (termed GOR-CRISPR) to create a simple colorimetric detection system for MTB (Figure 1). This approach involves three key steps. First, MTB DNA is released from bacterial cells and extracted using a specialized kit for RPA, ensuring that target DNA is available for the downstream reaction. Second, the one-pot RPA-CRISPR reaction is carried out in the presence of glycerol, which enhances the efficiency of the combined amplification and cleavage processes. Third, upon target recognition and Cas12a activation, the cleavage of the ssDNA reporter triggers the self-assembly of G4-hemin DNAzyme, producing a color change. In the absence of the target, no cleavage occurs and the nanozyme does not form, so no color change is observed. In this system, RPA amplification of the target sequence leads to the generation of double-stranded DNA containing the protospacer for Cas12a. Cas12a, guided by the crRNA, then recognizes the target and cleaves a single-stranded DNA probe (ssDNAR) that is present as a reporter. The ssDNAR was designed to be complementary to an ssDNA-hemin strand. When ssDNAR is cleaved by Cas12a, it can no longer hybridize to the ssDNA-hemin; instead, the fragments of ssDNAR combine with the ssDNA-hemin to form an active G4-hemin DNAzyme. This DNA nanozyme catalyzes the oxidation of TMB, turning the solution visibly blue. If the target DNA is absent, the intact ssDNAR will hybridize with the ssDNA-hemin to form a double-stranded structure, preventing G4 nanozyme formation and leaving the solution colorless. The entire detection process is completed in about 60 min. By leveraging Cas12a′s trans-cleavage property and the signal amplification from the G4-hemin DNAzyme, this strategy achieves enhanced sensitivity, efficiency, and accuracy in detecting MTB DNA.

In addition, this study conducted a feasibility analysis using a fluorescence system, targeting the MTB genomic DNA as the target, and using enzyme-free water as the template as the negative control. The results are shown in Supplementary Figure S1. When there was no targeted/no reporter guidance from the crRNA and no Cas12a protein cutting, almost no fluorescence signal was produced, and the detection results were basically consistent with the negative control; there was no significant difference compared to the negative control; only when all components of the RPA-CRISPR/Cas12a fluorescence system were complete, could a strong fluorescence signal be generated, and there was a significant difference compared to the control group (p < 0.005). The experiment indicates that the complete RPA-CRISPR/Cas12a system can accurately detect MTB, and this study is feasible.

### 3.2 Establishment of GOR-CRISPR method

**FIGURE 2:**
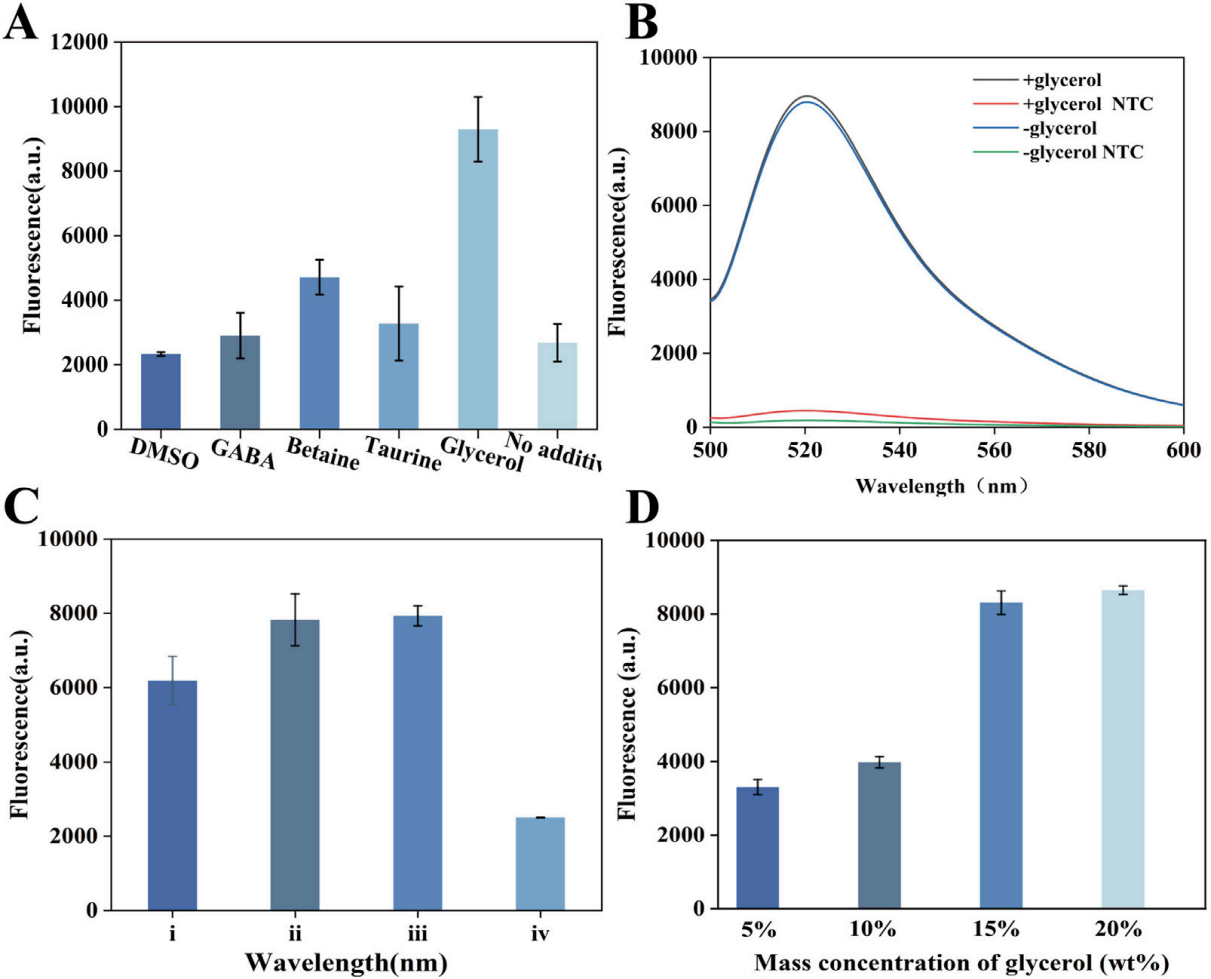
Establishment of GOR-CRISPR/Cas12a. **(A)** Analysis of the effect of various additives on one-pot RPA-CRISPR/Cas12a. **(B)** Effect of varying glycerol concentrations on RPA-CRISPR/Cas12a. **(C)** Effects of different positions of the three components. **(D)** Assessment of the effects of 15% glycerol on one-pot RPACRISPR/Cas12a system.

We first applied glycerol to optimize the one-pot GOR-CRISPR/Cas12a reaction. Previous studies have suggested that certain additives (e.g., DMSO, betaine) can improve nucleic acid amplification and detection efficiency (Henke et al., 1997; Li Z. et al., 2021). We evaluated various additives in our one-pot system (Figure 2A) and found that glycerol was uniquely effective in enhancing the reaction. To investigate the mechanism by which glycerol improves the one-pot RPA-CRISPR/Cas12a system, we examined whether glycerol promoted the CRISPR/Cas12a detection or the RPA reaction. Initially, the effect of glycerol on the CRISPR system was assessed using an isolated CRISPR/Cas12a assay, which showed that glycerol did not enhance the CRISPR reaction (Figure 2B). Additionally, we examined the effect of glycerol on RPA amplification. Interestingly, the electrophoretic bands revealed that glycerol only marginally inhibited RPA amplification (Supplementary Figure S2). At the same time, we used fluorescence to assess the effect of 15% glycerol on the one-pot RPA-CRISPR/Cas12a system (Supplementary Figure S3). We also examined the configuration of the reaction components within a single tube (Figure 2C). Corresponding to experiences i, where the mixtures of RPA and CRISPR system were on the bottom of tube and the glycerol was on top. Corresponding to experiences ii, where the RPA component was on the bottom and the mixtures of glycerol and CRISPR system were on top. Corresponding to experiences iii, where the mixtures of glycerol and CRISPR system were on the bottom of the tube and the RPA was on top. Corresponding to experiences iv, where the CRISPR system was on the bottom of the tube and the mixtures of RPA and glycerol were on top. In a setup without a glycerol separation layer (configuration i in Figure 2C), the CRISPR/Cas12a and RPA reactions interfered with each other, resulting in poor detection performance. By contrast, when we introduced a glycerol layer to spatially separate the RPA and CRISPR components (configurations ii and iii in Figure 2C), the detection signal improved significantly. We optimized the glycerol concentration and identified an optimal level that maximized the colorimetric signal (Figure 2C). We optimized the glycerol concentration using fluorescence detection and identified 15% as the optimal level, which maximized the colorimetric signal (Figure 2D).

### 3.3 G4-hemin DNAzyme color reaction

**FIGURE 3:**
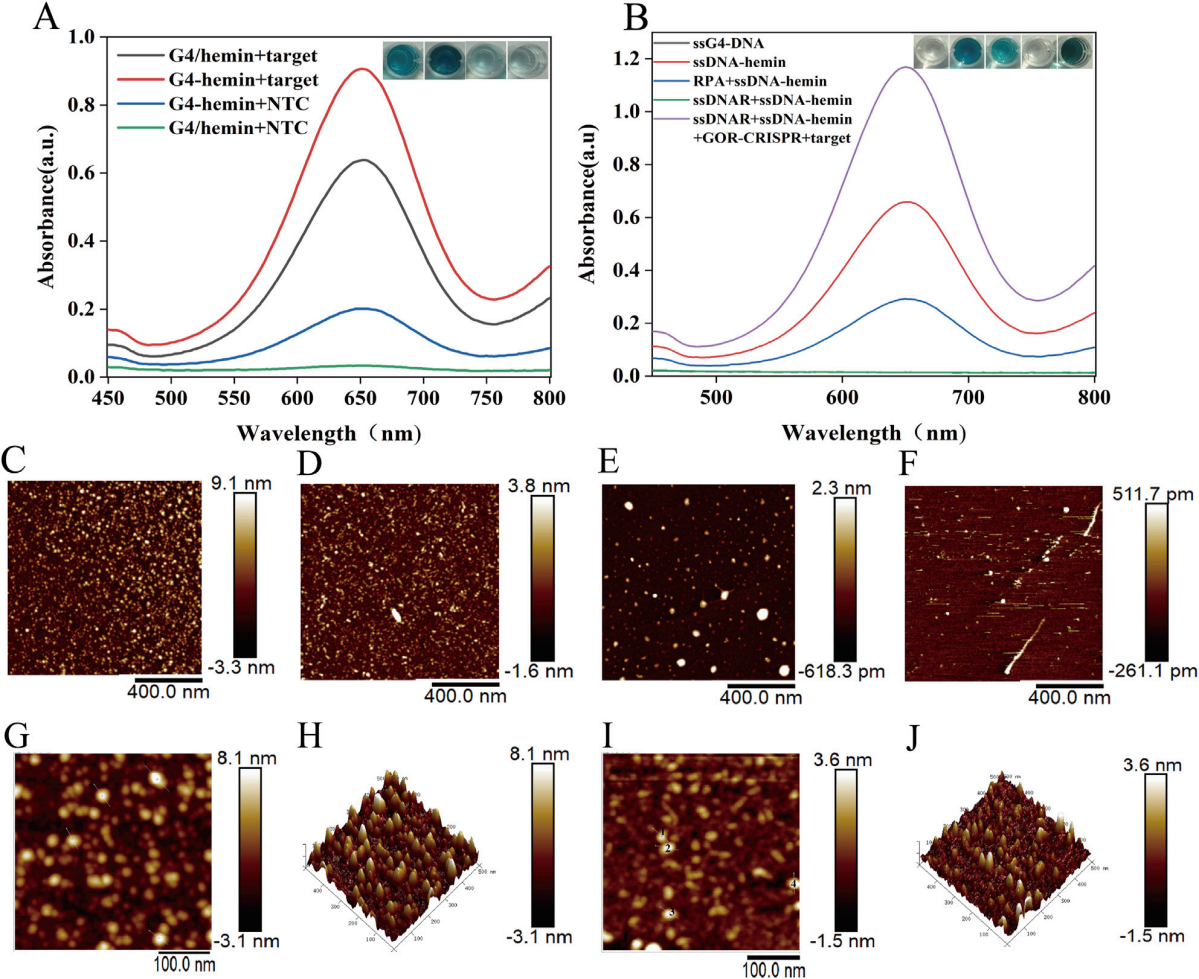
G4-hemin DNAzyme color reaction. **(A)** The color change and absorption spectra of TMB in various reaction systems. **(B)** The absorption spectra of G4-hemin and G4/hemin, as well as the color change of TMB, in various reaction systems. **(C)** AFM image of G4-hemin DNAzyme. **(D)** AFM image of dsDNA-hemin. **(E)** AFM image of ssDNA-hemin. **(F)** AFM image of ssDNAR. **(G)**, **(H)** HP-AFM image of G4-hemin DNAzyme. **(I)**, **(J)** HP-AFM image of dsDNA-hemin.

The oxidase-like activity of G4-hemin DNAzyme was assessed through the oxidation of TMB. As illustrated in Figure 3A, the CRISPR system activated by the target gene facilitated the formation of G4-hemin DNAzyme, evidenced by an increased absorption peak at 650 nm resulting from TMB oxidation and demonstrating excellent oxidase-like activity. Furthermore, the oxidation activity of the self-assembled G4-hemin DNAzyme developed in this study surpasses that of traditional G4/hemin systems. Figure 3B presents the oxidative activity of the relevant solutions tested in this study using TMB. The synthesized ssDNA-hemin exhibited oxidative activity, whereas the RPA + ssDNA-hemin demonstrated diminished oxidative activity. The ssDNAR + ssDNA-hemin combination, due to the complementary nature of ssDNAR to ssDNA in ssDNA-hemin, forms a duplex that does not facilitate specific G4 formation, resulting in a significant reduction of the absorption peak. This observation is markedly different from that of ssDNAR + ssDNA-hemin + GOR + target, underscoring the high feasibility of this study. These findings confirm the successful assembly of the G4-hemin nanozyme. UV-vis absorption measurements further validated that only the combination of cleaved ssDNAR and ssDNA-hemin (in the presence of the complete GOR-CRISPR system and target DNA) led to effective TMB oxidation. Collectively, these optimizations established the one-pot GOR-CRISPR/Cas12a reaction, integrating G4-hemin DNAzyme generation as a highly sensitive detection method.

In addition, we confirmed the formation of the G4-hemin DNAzyme using atomic force microscopy (AFM). The AFM image of the assembled G4-hemin DNAzyme revealed distinct nanostructures, while control samples-including dsDNA-hemin, ssDNA-hemin, and ssDNAR-exhibited different morphological characteristics (Figures 3C–F). Figure 3C indicates that the G4-DNA-hemin complex forms highly stacked structures, measuring 9.1 nm, which supports the hypothesis that Hemin induces G4-DNA self-assembly and provides a structural basis for the application of G4-DNA in nanocatalysis and biosensing. The AFM results in Figure 3D demonstrate that Hemin binds to dsDNA to form a local bulge of 3.8 nm, representing a multimolecular complex. Additionally, the AFM data in Figure 3E show that Hemin binds to ssDNA to form a local height of 2.3 nm, which, when compared with G4-DNA-Hemin complexes reported in the literature (∼2–4 nm), indicates that ssDNA also binds Hemin efficiently and exhibits trace enzyme-like catalytic activity. Finally, Figure 3F successfully presents AFM imaging of single-stranded DNA, revealing a feature height of 0.5117 nm and a local depression of −0.2611 nm. The self-assembled G4-DNA-hemin nanozymes and ds-DNA-hemin were further analyzed using high-power atomic force microscopy (HP-AFM) (Figures 3G–J). The tapping-mode HP-AFM image (Figures 3G,H) reveals discrete assembly units of G4-DNA-hemin complexes with an average height of (8.1 ± 0.7) nm, alongside subsurface defect structures with a depth of (3.1 ± 0.9) nm. This image provides direct visual evidence for the structural reorganization of G4-DNA. The HP-AFM image (Figures 3I,J) distinctly illustrates the nanoscale structural features of the dsDNA-hemin complex: a protrusion measuring (3.6 nm), which significantly exceeds the canonical B-form dsDNA diameter (∼2 nm), and a depression of approximately (1.5 nm). The G4-hemin DNAzyme complex forms a multilayered structure (8.1 nm protrusion) through porphyrin ring stacking, exhibiting enhanced peroxidase-mimicking activity, which is consistent with theoretical predictions.

### 3.4 Optimization of experimental conditions

**FIGURE 4:**
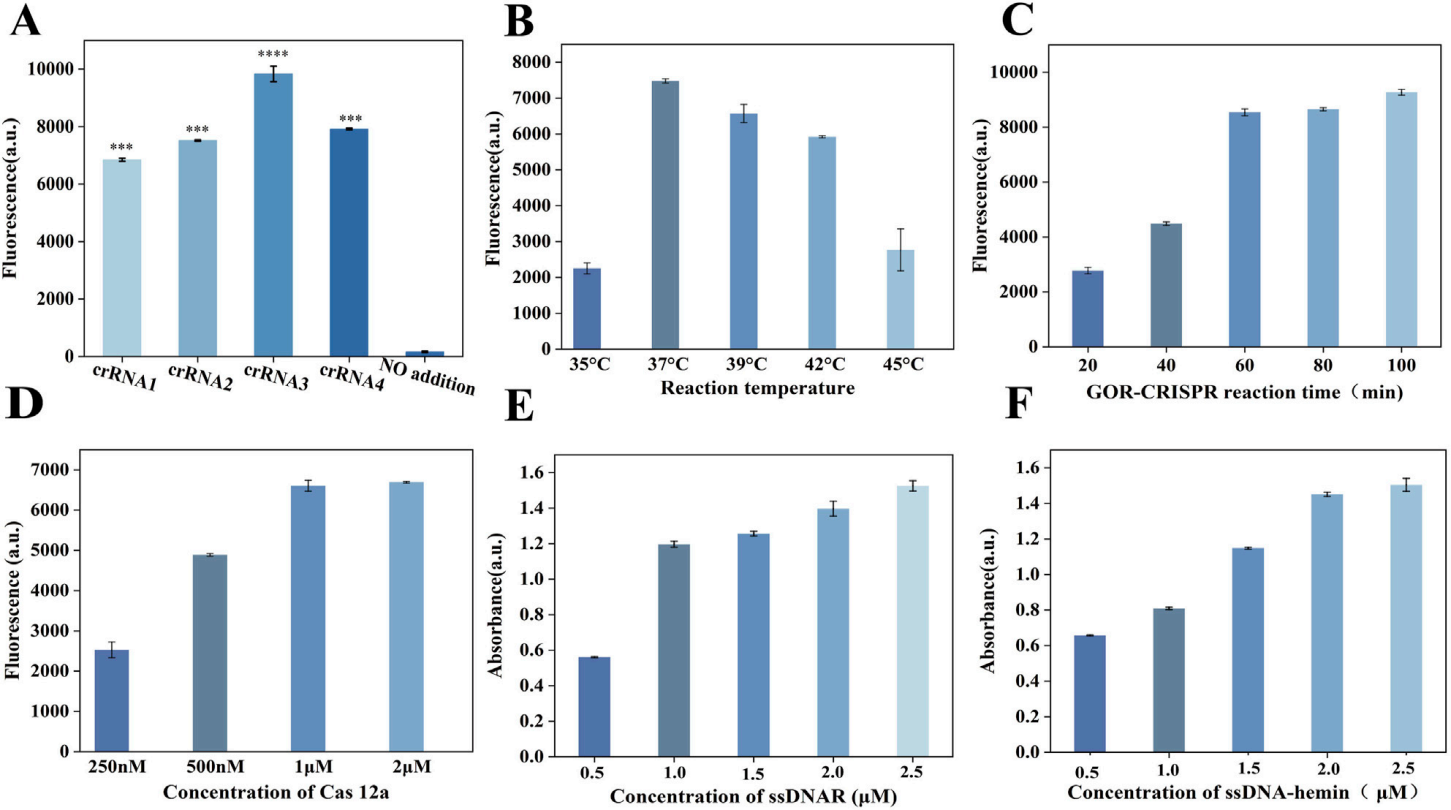
Optimization of the GOR-CRISPR reaction conditions and G4-hemin DNAzyme visualization system. **(A)** Fluorescence intensity readings after 30 min for four distinct crRNAs in the RPA-CRISPR/Cas12a reaction. **(B)** Reaction temperature optimization (35, 37, 39, 42, 45°C). **(C)** Reaction time optimization (20, 40, 60, 80, 100 min). **(D)** Cas12a concentration optimization (0.25, 0.5, 1 and 2 μM). **(E)** ssDNAR concentration optimization (0.5, 1, 1.5, 2, and 2.5 μM). **(F)** ssDNA-hemin concentration optimization (0.5, 1, 1.5, 2, and 2.5 μM).

We designed three sets of RPA primers and four crRNAs for the assay. To identify the most effective primers, we tested each set in the RPA reaction. Polyacrylamide gel electrophoresis analysis (Supplementary Figure S4) showed that the primer pair F1/R1 produced the strongest amplification product, indicating the highest amplification efficiency. The plasmid solution with known copy number was serially diluted to 10^5^–10^0^ copies/µL, and then subjected to PAGE after RPA amplification under the same conditions (Supplementary Figure S5). For crRNA selection, we conducted a fluorescence-based CRISPR-Cas12a cleavage assay using *in vitro* transcribed target DNA. As shown in Figure 4A, crRNA3 generated the most robust fluorescence signal, reflecting the most efficient Cas12a activation kinetics. Based on these results, we selected the RPA primer pair F1/R1 and crRNA3 for our one-pot RPA-CRISPR/Cas12a detection system. Enzymatic reactions utilize DNA nanozymes in conjunction with glycerol-enhanced one-pot RPA-CRISPR/Cas12a. The detection capacity is significantly influenced by several factors, including reaction time, temperature, buffer composition, enzyme concentration, and Cas12a protein concentration. Various conditions affecting endpoint fluorescence intensity were systematically optimized and evaluated to achieve optimal detection results. To determine the optimal incubation temperature during our detection process, we selected 35°C–45°C, which is the routine incubation temperature range of proteins. We found that the fluorescence signal was the most intense at 37°C (Figure 4B). Therefore, the optimal temperature for our detection was 37°C. To determine the optimal incubation time, the sample was incubated for 20–100 min. The fluorescence signal intensity increased significantly at 60 min, but the change was not obvious at 80 min and 100 min (Figure 4C). Therefore, all subsequent experiments were conducted for 60 min. In addition, three common buffers (NEBuffer 2.1r, 3.1r, and rCutSmart) were evaluated, among which the 2.1r buffer was determined to be the best choice (Supplementary Figure S6). Primer concentration significantly influenced the amplification intensity. The optimal primer concentration was determined to be within the range of 0–0.72 µM (Supplementary Figure S7), with the highest signal intensity in the fluorescence spectrum occurring at 0.24 µM. The cleavage reaction rate depends on the amounts of the Cas enzyme, making their concentrations critical for this process.We examined the Cas enzyme concentration within the range of 0.25–2 μM, observing that the fluorescence signal remained consistent when the concentration of Cas12a exceeded 1 µM (Figure 4D); therefore, we used 1 µM Cas12a protease in our experiments. Furthermore, the concentration of the reaction enzyme, MgOAc, is highly important for the whole detection. Experiments were performed at concentrations ranging from 0 to 56 mM. By evaluating the fluorescence intensity (Supplementary Figure S8), the catalytic effect was found to be optimal at 28 mM, so this concentration was selected for subsequent experiments. ssDNAR was detected in the concentration range of 0.5–2.5 µM, and the fluorescence intensity (Figure 4E) was strongest at 2 µM. The ssDNA-hemin concentration was also crucial to the color reaction, so we screened concentrations in the range of 0.5–2.5 µM and monitored the reaction using UV-vis spectroscopy (Figure 4F). The optimal concentration was determined to be 2 µM.

### 3.5 Assessment of the analytical sensitivity

**FIGURE 5:**
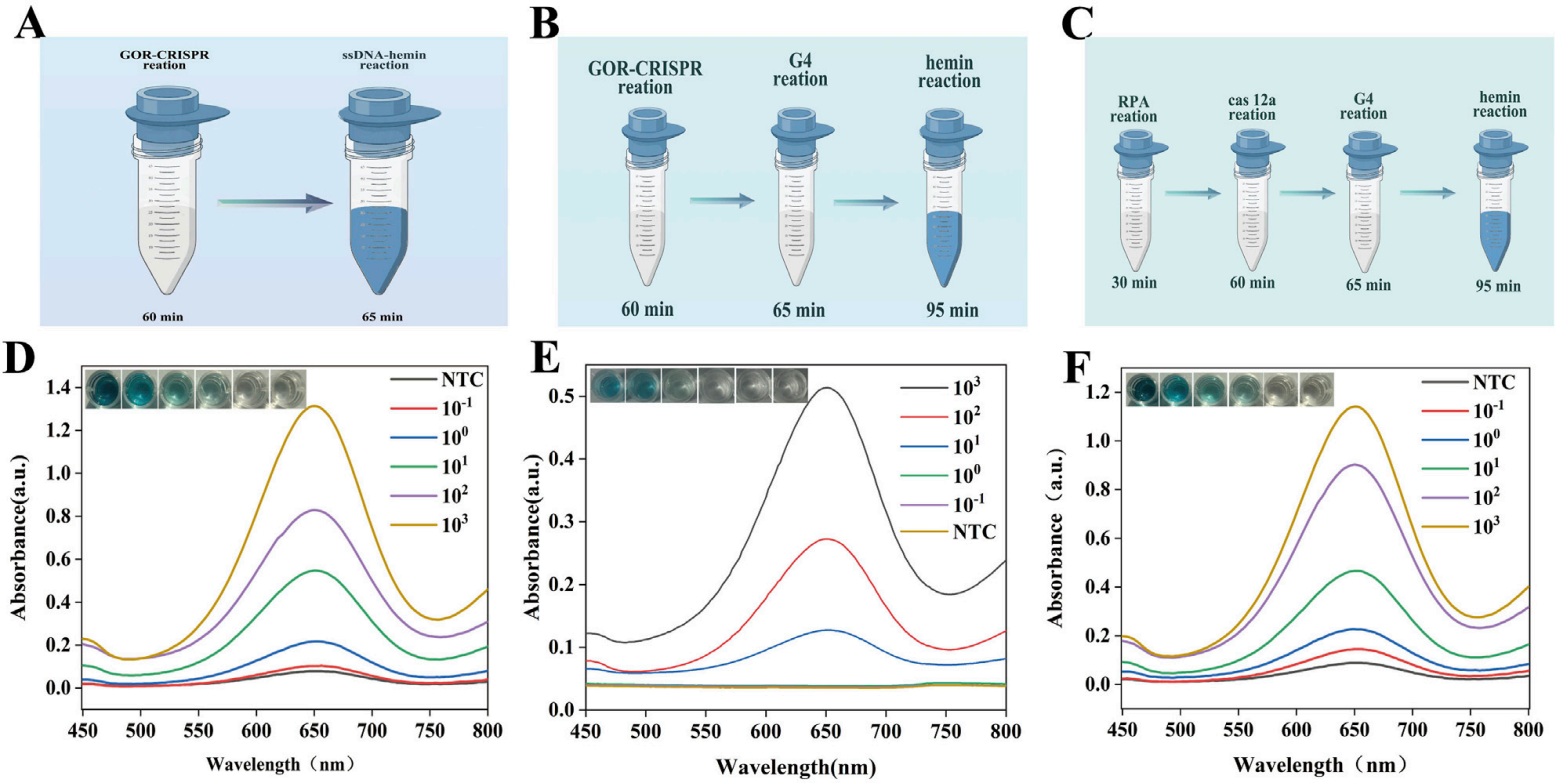
Analytical sensitivity analysis of GOR-CRISPR reaction with G4-hemin DNAzyme visualization system. **(A)** Diagram of the GOR-CRISPR reaction with G4-hemin DNAzyme visualization system. **(B)** Diagram of the GOR-CRISPR reaction and traditional G4- DNAzyme process. **(C)** Diagram of the traditional four-stage process. **(D)** Absorption spectra for varying DNA concentrations using GOR-CRISPR reaction with G4-hemin DNAzyme visualization system. **(E)** Absorption spectra for varying DNA concentrations using the GOR-CRISPR reaction with traditional G4-DNAzyme process. **(F)** Absorption spectra for varying DNA concentrations using the traditional four-stage approach. NTC represents no target control.

The sensitivity of the assay was determined using dilute MTB DNA (Figures 5A,D). The color change of the detection result is directly related to the target fragment concentration and was monitored via UV-vis absorption spectroscopy and mapping. The minimum detection limit was 10 copies/μL. We compared the sensitivity of the GOR CRISPR reaction to that of the traditional G4-DNAzyme process (Figures 5B,E). The result indicated that this adaptation resulted in a slightly lower detection sensitivity of 10^2^ copies/μL and exhibited suboptimal detection efficiency. Additionally, traditional RPA, CRISPR, and G4-DNA nanozyme assays typically require four distinct steps to complete the entire detection process. The workflow of these conventional multi-step assays is summarized in Figures 5C,F. The UV-vis absorption spectrum indicates that the minimum detection sensitivity achievable using these conventional methods is 10 copies/μL. Consequently, the development of a one-pot method utilizing glycerol to enhance the reaction, in conjunction with a specifically designed G4-hemin DNAzyme, is highly justified. This new approach not only simplifies the detection process but also provides analytical sensitivity comparable to that of traditional four-step methods.

### 3.6 Clinical performance evaluation of biosensor for TB detection

**FIGURE 6:**
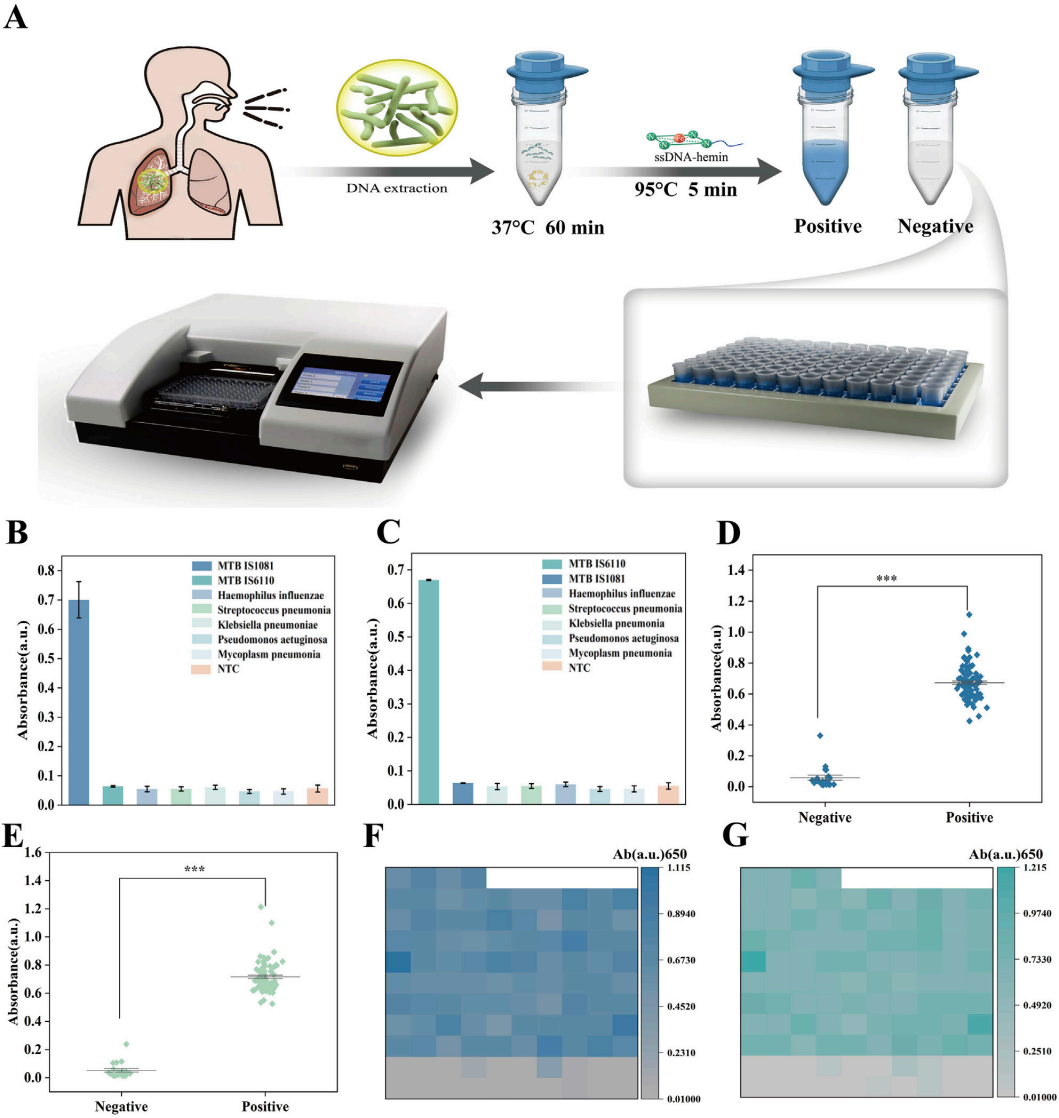
**(A)** MTB assay by GOR-CRISPR with G4-hemin DNAzyme approach. **(B)** Specificity analysis of MTB IS1081 detection by UV-vis absorption analysis. **(C)** Specificity analysis of MTB IS6110 detection by UV-vis absorption analysis. **(D)** Absorbance intensity scatter plot for 20 negative and 84 positive samples. **(E)** Absorbance intensity scatter plot of 20 negative and 84 positive samples. **(F)** Heat map showing the MTB IS1081 detection results in 84 positive samples and 20 negative samples. **(G)** Heat map showing the MTB IS6110 detection results in 84 positive samples and 20 negative samples. The data are presented as the mean ± s. **(D)** for three technical replicates.

We combined the catalytic activity of G4-hemin DNAzyme with the glycerol-enhanced one-pot RPA-CRISPR/Cas12a reaction to create a simple colorimetric detection system for *Mycobacterium tuberculosis* (MTB) (Figure 6A). The first step was to extract DNA from patient sputum samples. The second step was to conduct a glycerol-promoted one-pot RPA-CRISPR reaction that could enhance the efficiency of the combined amplification and cleavage process. When the target was detected and Cas12a was activated, the cleavage of the single-stranded DNA signal would trigger the self-assembly of the G4-heme DNA nanozyme at 95°C, resulting in a color change. If there was no target, no cleavage would occur and the nanozyme would not form, thus no color change would be observed. In this system, the complementary relationship between the single-stranded DNA probe (ssDNAR) that acts as the reporter for Cas12a to recognize and cleave and the single-stranded DNA-hemoglobin chain forms at 95°C. At this time, the spatial structure of the cas12a protein is disrupted, its cleavage function is lost, and there will be no situation of continuous cleavage by the G4-heme DNA nanozyme. To assess the specificity of the GOR-CRISPR with G4-hemin DNAzyme system in target gene detection, this study obtained two samples derived from the genes of *Mycobacterium tuberculosis*: IS1081 and IS6110. The IS6110 region has been confirmed to have a high sensitivity in diagnosing tuberculosis using polymerase chain reaction (PCR), and the copy number in each genome can reach up to 25. However, at the same time, there are descriptions stating that there are *Mycobacterium tuberculosis* isolates without the target sequence of IS6110. To ensure coverage of all *Mycobacterium tuberculosis* species, this study also adopted the sequence IS1081. Unlike IS6110, IS1081 exists in all *Mycobacterium tuberculosis* species, and the copy number in each genome is stable at 5-7 repeated sequences. Additionally, five respiratory samples from clinically diagnosed single pathogen infections (i.e., *Haemophilus influenzae, Streptococcus pneumoniae, Klebsiella pneumoniae, Pseudomonas aeruginosa, and Mycoplasma pneumoniae*) were collected for MTB DNA detection. The results demonstrated that apart from a positive reaction in the detection of the MTB gene IS1081, the remaining six samples all exhibited negative reactions (Figure 6B). And Figure 6C shows that, except for the sample that showed a positive reaction for the tuberculosis *bacillus* gene IS6110, the other six samples all showed negative reactions. The specific results of genes 1801 and 6,110 were verified by fluorescence, as shown in Supplementary Figures S9,S10.

To further evaluate the performance of the biosensing system in clinical sample detection, 104 sputum samples were collected from patients suspected of tuberculosis infection in clinical settings. These samples were detected using the GOR-CRISPR G4-hemin DNAzyme system (Figures 6D,E), and qRT-PCR was employed as a reference method (with each sample undergoing parallel detection three times) (Supplementary Figures S11,S12). The results showed that all 84 MTB positive samples identified by qRT-PCR were also positive when detected with the GOR-CRISPR G4-hemin DNAzyme system (positive coincidence rate: 100%). Similarly, all 20 MTB negative samples detected by qRT-PCR were negative using the GOR-CRISPR G4-hemin DNAzyme system (negative coincidence rate: 100%). In conclusion, the biosensing system presents multiple advantages that including high sensitivity, good specificity, rapid detection speed, low detection cost, and independence from specialized instrumentation. As such, it holds great promise for future rapid POCT. Moreover, heatmap analysis of the results for MTB IS1081 and IS6110 (Figures 6F,G) revealed significant differences between positive and negative samples, with no classification errors. These findings suggest that the biosensing system features high sensitivity and excellent specificity in detecting the MTB target gene IS1081.

## 4 Conclusion

In summary, we have developed an ultrasensitive naked-eye biosensing system for MTB detection that integrates a one-pot RPA-CRISPR/Cas12a reaction with G4-hemin DNAzyme self-assembly. The inclusion of glycerol as a separation barrier significantly improves compatibility between the RPA amplification and the CRISPR/Cas12a trans-cleavage process, enabling both to function optimally in a single tube. The CRISPR/Cas12a-mediated cleavage of the ssDNA reporter and its subsequent self-assembly with an ssDNA-hemin strand into G4-hemin nanozymes occur seamlessly within the one-pot system, greatly enhancing the catalytic signal output. Notably, this simple assay can achieve rapid (<60 min), low-cost, and highly sensitive detection of MTB without the need for specialized equipment. Moreover, the platform is easily adaptable to detect other pathogens or genetic targets by changing the sequence of the guide RNA and reporter, demonstrating considerable potential as a universal point-of-care diagnostic technology.

## Data Availability

The original contributions presented in the study are included in the article/Supplementary Material, further inquiries can be directed to the corresponding authors.
