# Supplementary material for: A naked-eye biosensing system based on one-pot RPA-CRISPR/Cas12a driver G4-hemin self-assembly for Mycobacterium tuberculosis
Source: Front Chem. 2025 Aug 7;13:1631086. doi: 10.3389/fchem.2025.1631086 (PMC12367758; doi:10.3389/fchem.2025.1631086)
Supplement: Supplementary file 1 [file DataSheet1.pdf]

Supplementary information contents

Table S1 Nucleic acids sequences used in this work.

Name	Sequence
RPA-F1	CCAAGCTGCGCCAGGGCAGCTATTTCCCGGAC
RPA-F2	CAAGCGAGCTGAACGCGCACTGACCAGCGT
RPA-F3	GGTGGCGACCTGCTACCTGCTGGGAGTAT
RPA-R1	TTGGCCATGATCGACACTTGCGACTTGGA
RPA-R2	CGAAACGCCTCTACGGCTTCGTCGAGCTC
RPA-R3	GGCGTCGGCGGCGAGGAAGGTATACGG
crRNA1	UAAUUUCUACUAAGUGUAGAUCCGGACUGGCUGCUGCAGCG
crRNA2	UAAUUUCUACUAAGUGUAGAUGACACCCGUGCCGCAACCAU
crRNA3	UAAUUUCUACUAAGUGUAGAUUCACCGACGCCUACGCUCGC
crRNA4	UAAUUUCUACUAAGUGUAGAUCAAGUCGCAAGUGUCGAUCAUG
ssDNAR-FQ	FAM-TTTCCCAACCCGCCCTACCCA-BHQ1
ssDNA-hemin	Hemin-TGGGTAGGGCGGGTTGGGAAA
ssDNAR	TTTCCCAACCCGCCCTACCCA
ssDNA	TGGGTAGGGCGGGTTGGGAAA

Supporting Figures

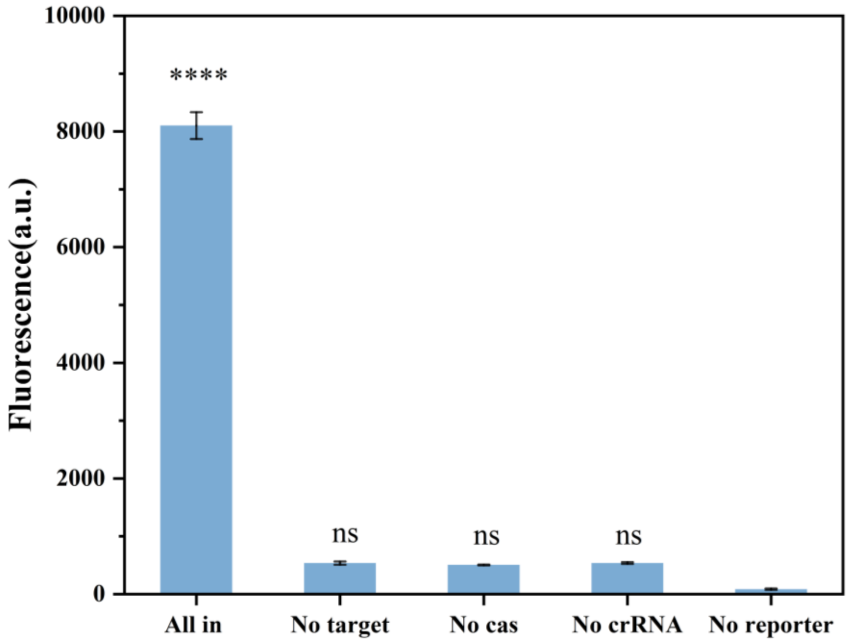

Fig. S1 Fluorescence intensity at 60 minutes of reaction for different component systems

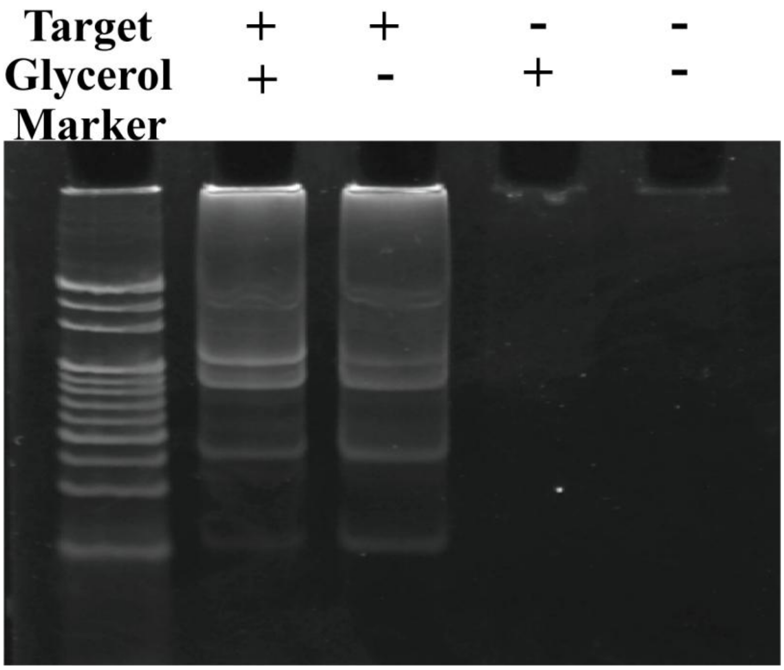

Fig. S2 PAGE Gel electrophoresis analysis of the effects of 15% glycerol on RPA reaction

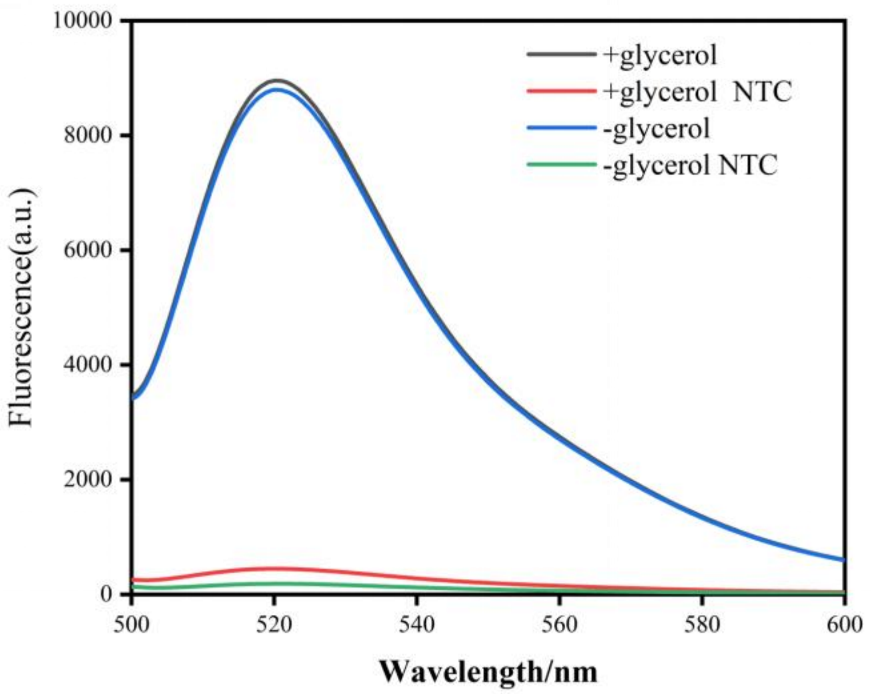

Fig. S3 Assessment of the effects of 15% glycerol on one-pot RPA-CRISPR/Cas12a system

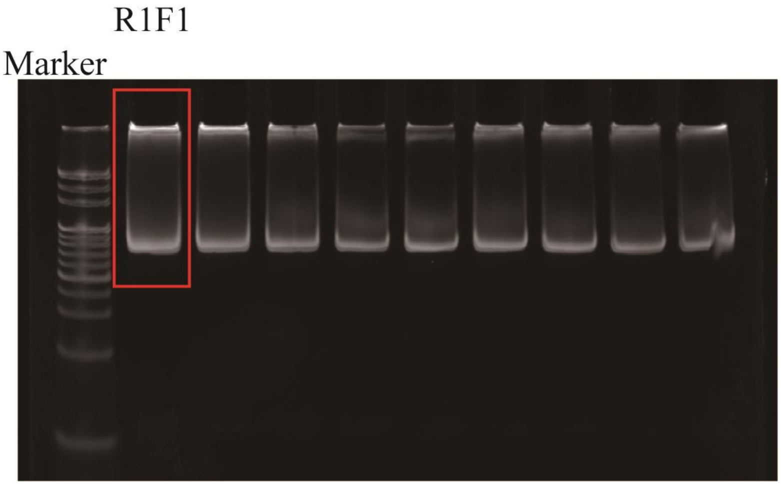

Fig. S4 PAGE Gel electrophoresis analysis of different primers on RPA reaction

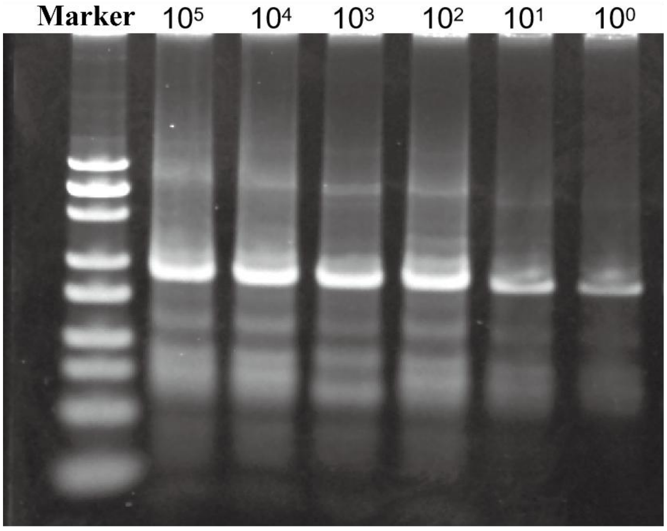

Fig. S5 PAGE Gel electrophoresis analysis of different concentrations（10^5 - 10^0 copies/μL） in the RPA reaction

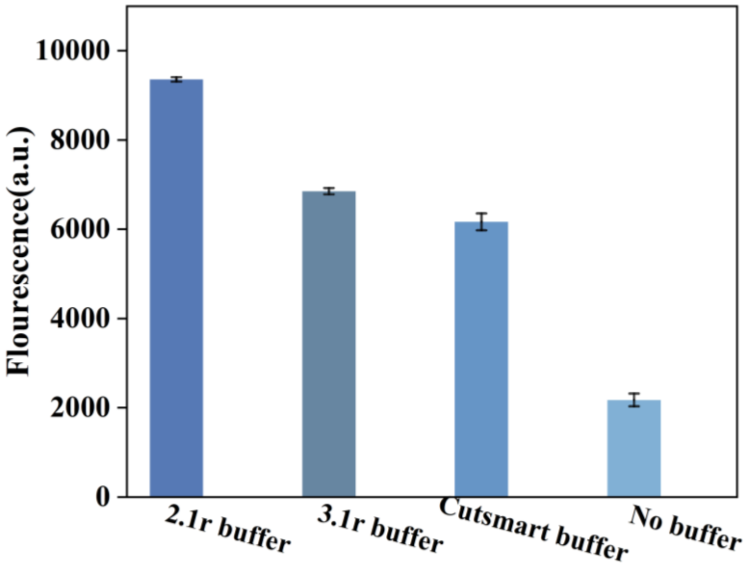

Fig. S6 Screening of reaction buffer solutions

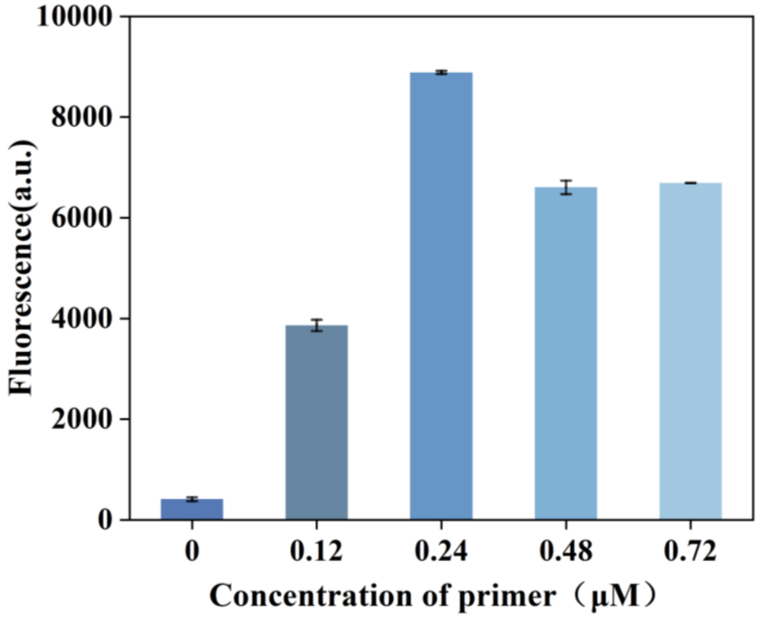

Fig. S7 Primer concentration optimization

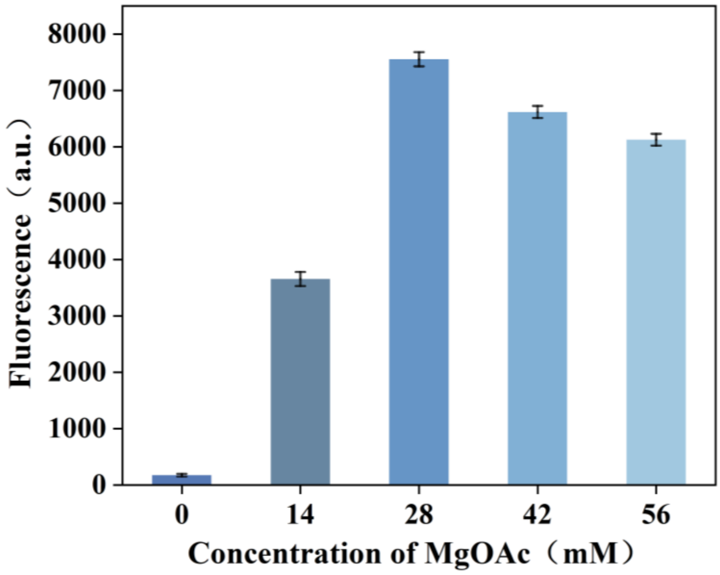

Fig. S8 MgOAc concentration optimization

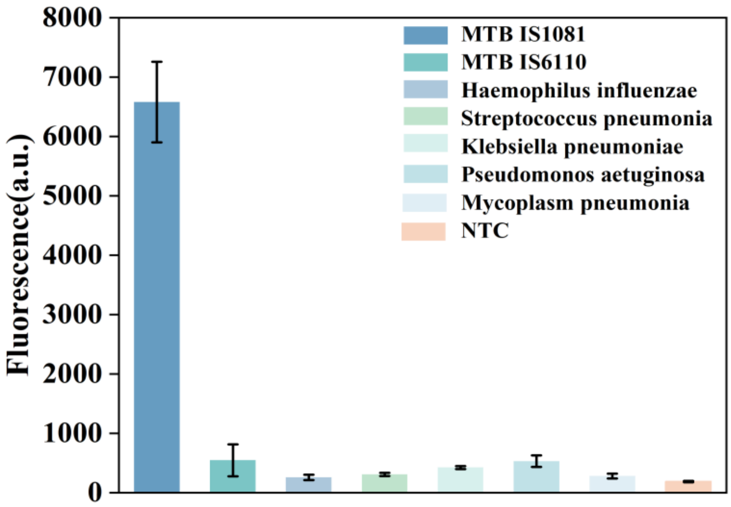

Fig. S9 Specificity analysis of MTB IS1081 detection by fluorescence intensity analysis

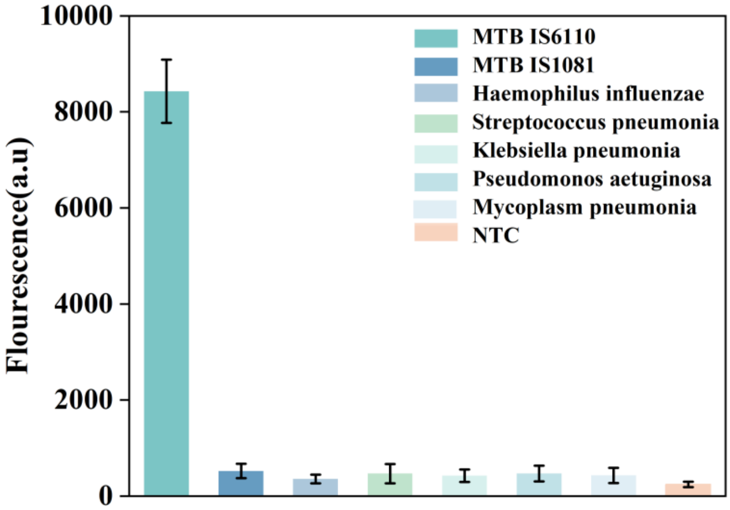

Fig. S10 Specificity analysis of MTB IS6110 detectionby fluorescence intensity analysis

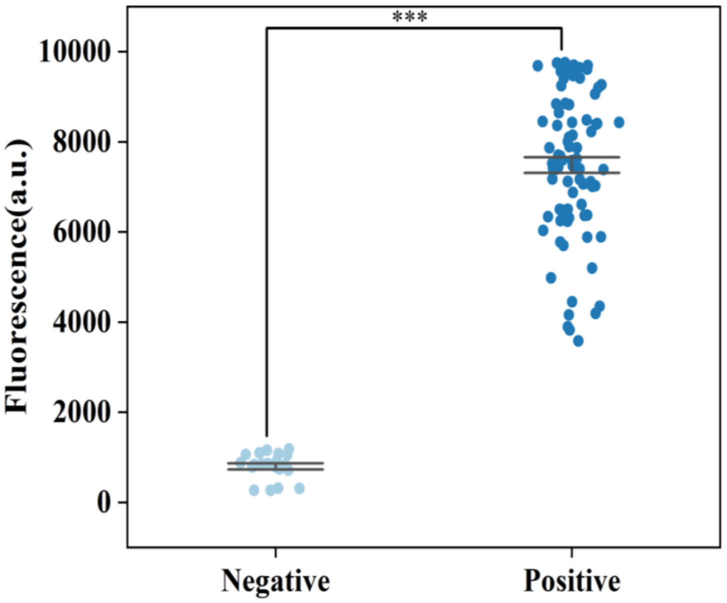

Fig. S11 Fluorescence intensity scatter diagram for 20 negative and 84 positive samples

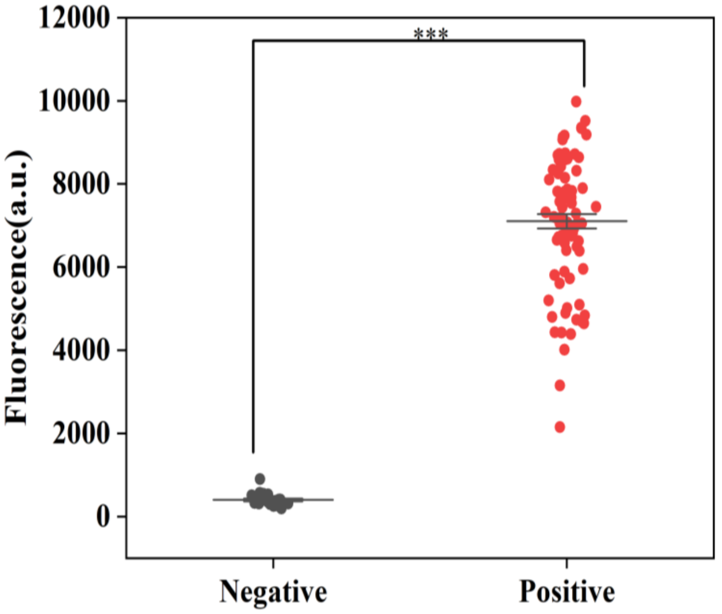

Fig. S12 Fluorescence intensity scatter plot of 20 negative and 84 positive samples
